# Human Endogenous Retroviruses in Myalgic Encephalomyelitis/Chronic Fatigue Syndrome: Emerging Roles in Pathogenesis, Immunity, Biomarkers and Therapeutics

**DOI:** 10.3390/ijms27104309

**Published:** 2026-05-12

**Authors:** Krishani Dinali Perera, Elisa Oltra, Simon R. Carding

**Affiliations:** 1 Food, Microbiome and Health Research Programme, Quadram Institute Bioscience, Norwich Research Park, Norwich NR4 7UQ, UK; krishani.perera@quadram.ac.uk; 2 Department of Pathology, School of Health Sciences, Universidad Catolica de Valencia, 46001 Valencia, Spain; 3 Norwich Medical School, University East Anglia, Norwich NR4 7UQ, UK

**Keywords:** ME/CFS, HERVs, immunity, biomarker, patients, classification

## Abstract

Human endogenous retroviruses (HERVs) are potential driving forces of the pathophysiology of Myalgic encephalomyelitis/chronic fatigue syndrome (ME/CFS), linking post-infectious immune dysfunction to chronic inflammation and immune and neurocognitive dysfunction that are hallmark features of ME/CFS. Accumulating evidence from related autoimmune diseases and cancers has shown that reactivated HERVs can contribute to disease pathogenesis by amplifying immune activation through viral protein-mediated innate sensing, long terminal repeat (LTR)-driven transcription, and disrupting epigenetic silencing. HERV signatures are therefore promising biomarkers for diagnosis, patient stratification for drug-repurposing trials, and therapy monitoring. Accumulating evidence suggests a possible correlation between HERV expression and ME/CFS symptom severity, alterations in immune phenotypes, function and inflammatory gene networks. Importantly, locus-specific HERV profiling is a promising approach for distinguishing ME/CFS from overlapping or co-morbid conditions and healthy controls. Furthermore, HERV-targeted antibodies, immune modulators, epigenetic and antiviral interventions offer promise as concomitant therapeutic strategies for ME/CFS. Additional research incorporating viromics and other-omics validation, functional assays, and HERV-stratified clinical trials is now needed to realise this potential and to transform ME/CFS from a symptom-based syndrome into a mechanism-driven, treatable condition.

## 1. Introduction

Myalgic encephalomyelitis/chronic fatigue syndrome (ME/CFS) is a complex, heterogeneous, and debilitating illness characterised by multi-organ dysregulation that compromises cellular energy production and metabolism, leading to a wide range of symptoms and impaired physical and cognitive function [1,2]. The most common patient-reported symptoms include post-exertional malaise (PEM), cognitive impairment, persistent fatigue, unrefreshing sleep, immune and gastrointestinal dysfunction, flu-like symptoms, and a general reduction in energy levels [3]. ME/CFS is not a sex-restricted condition, but it is more frequently reported in females [4,5,6,7,8], with its prevalence varying significantly across different age groups [6,7,8]. Epidemiological studies further suggest that ME/CFS is diagnosed more often among non-Hispanic white populations compared to those of other ethnic backgrounds, including Black or Asian groups [6,7]. In the UK alone, approximately 0.6% of the population appears to be affected by ME/CFS [6], and globally, the condition could impact up to 70 million people [9]. Nevertheless, factors such as socioeconomic status, healthcare access, disease awareness, cultural stigma, and underreporting contribute to delays and discrepancies in diagnosis and data collection.

Although ME/CFS has diverse aetiological factors, infection has been identified as the trigger for 50% or more of ME/CFS sufferers [3]. However, no single or specific pathogen has yet been identified, with various viruses, bacteria, and fungi being associated with the onset or perpetuation of ME/CFS symptoms. Of these, virus infections are considered the most likely infectious trigger, with many patients reporting the onset of symptoms following a flu-like illness, initiated by an acute viral infection (e.g., SARS-CoV-2, influenza virus) or chronic infection and reactivation of latent viruses (e.g., Epstein–Barr virus or EBV, human herpesviruses or HHVs, enterovirus) [10,11,12]. Emerging evidence from transcriptomic, proteomic, and serological studies has highlighted a possible contribution by Human Endogenous Retroviruses (HERVs) to disease pathogenesis, with HERV transcriptomes in immune cells and tissues of ME/CFS patients differentiating them from healthy controls and from patients with fibromyalgia (FM) [13]. This review provides an overview of HERV biology and discusses the current findings from published studies investigating HERV expression in ME/CFS and HERV-related pathophysiological mechanisms that may drive the disease. We also discuss the implications these findings might have on the diagnosis and treatment of ME/CFS.

## 2. HERV Biology

### 2.1. HERVs

HERVs are viral “fossils” in the human genome, originating from exogenous retroviral infections that integrated into the mammalian germline cells, with many HERV families becoming fixed during the evolution of primate ancestors millions of years ago. Today, HERV sequences make up 8% of the human genome and are transmitted vertically in the population [14,15,16]. The integrated proviral HERVs contain internal viral coding regions: gag (group-specific antigen), pro (protease), pol (polymerase), and env (envelope), flanked by long terminal repeats (LTRs), that mimic the integrated forms of other exogenous retroviruses. However, many of these integrated proviruses can, over time, accumulate mutations and deletions through multiple recombination events, rendering them silent and non-infectious. As a result, no naturally occurring autonomously replicating HERV has been identified to date [17,18,19]. Most HERVs often lack one or more essential coding sequences, such as the env gene, but retain LTRs, and these often exist as solo LTRs [18,19,20,21,22]. Consequently, HERVs are classified as a subclass of transposable elements (TEs), which comprise approximately 45% of the human genome, and consist of DNA transposons and retrotransposons [15]. HERVs specifically belong to the LTR retrotransposon family, distinguishing them from non-LTR retrotransposons, such as long interspersed nuclear elements (LINEs) and short interspersed nuclear elements (SINEs).

### 2.2. HERV Classification

HERVs can be classified by the tRNAs that initiate reverse transcription at their primer binding sites, such as lysine tRNA for most HERV-K elements, or based on the nearby genes or characteristic amino acid motifs (for example, HERV-FRD) [23,24]. HERVs are grouped into three major classes based on their phylogenetic relatedness to exogenous retroviruses: class I gamma/epsilon-like viruses (HERV-H, -W, -E), class II alpha/beta/delta-like viruses (mainly HERV-K), and class III spumavirus-like viruses (HERV-L and -S) [22,23,24,25]. Class I contains the largest and mostly defective set of HERV families [24,26] while class II represents the second largest group, dominated by HERV-K [27]. Within class II, HERV-K elements are further classified into HML-1 to HML-11 subgroups, with HML-2 representing the most recent, best preserved integrations, mainly comprising intact envelope genes [27]. Most HML-2 proviruses are further classified into type 2 (intact proviruses that encode a nuclear export factor Rec, and a fusion-competent env glycoprotein) and type 1 (possessing a 292 bp pol-env deletion that abolishes canonical splicing for Rec/env proteins but produces a nuclear Np9 protein) [28,29]. However, some HML-2 loci can have larger deletions, evading this binary classification [27]. Class III elements are the least numerous and cluster with, yet remain distinct from, true spumaviruses [24].

### 2.3. HERV Transcription and Regulation

Expression of HERVs and their LTRs is tightly regulated by transcriptional silencing, either through evolutionary mechanisms such as the accumulation of point mutations and deletions or through epigenetic mechanisms. Nevertheless, low basal levels of HERV/LTR expression are observed across various cell types and tissues. This expression appears to be regulated by cell-specific epigenetic mechanisms and transcriptional regulatory elements that change throughout the human lifespan [30,31,32,33]. Thus, basal HERV expression levels vary across tissue types and may correlate with biological factors such as sex, ethnicity, and age, thereby collectively shaping the HERV transcriptome. Investigations of age-associated expression of HERV families revealed that several proviruses are moderately affected, leading to age-dependent expression profiles [34]. Thus, de-repression of HERV-K and other endogenous retroviruses in ageing and senescent human cells may be an active process that drives and amplifies cellular senescence and tissue ageing. This occurs partly through the production of retrovirus-like particles (RVLPs) that activate innate immune pathways (such as cyclic GMP-AMP synthase—stimulator of interferon genes or cGAS–STING pathway), propagating senescence to neighbouring cells [35]. Interestingly, drug-mediated inhibition could potentially alleviate this senescence and age-related tissue degeneration [35].

Basal HERV transcription is regulated primarily by two mechanisms: epigenetic control and so-called “leaky” expression. Epigenetic regulation of HERVs and their LTRs is essential for cellular homeostasis, with controlled HERV/LTR expression in the germline but not in somatic tissues, occurring through coordinated epigenetic mechanisms [36,37]. These tight epigenetic regulatory mechanisms include DNA methylation at CpG-rich LTR promoter regions, repressive histone markers such as H3K9 trimethylation (H3K9me3), and the activity of chromatin re-modellers, including SET domain bifurcated histone lysine methyltransferase 1 (SETDB1) and tripartite motif–containing 28 (TRIM28 or KAP1) [38]. Among these, DNA methylation has been extensively investigated as a key epigenetic regulatory mechanism for repressing HERV/LTR transcription. Differential methylation of HERV LTRs governs cell-type-specific transcriptional activity [39], exemplified by placental expression of syncytin—fusogenic env glycoproteins encoded by *ERVW-1* (syncytin-1) and *ERVFRD-1* (syncytin-2), which is tightly regulated by DNA methylation and histone modifications at their 5′ LTRs [40]. In some contexts, DNA hypomethylation of HERV LTRs is associated with tissue-specific enhancer activity [39,41]. However, loss of epigenetic silencing of HERV/LTRs could preferentially reactivate evolutionarily younger HERVs, such as HERV-K (HML-2), driving locus-specific transcription and dysregulation of nearby immune genes by acting as enhancers or alternative promoters [36,39,40,42]. In contrast, HERVs can also be transcribed autonomously when their 5′ LTR promoters escape epigenetic silencing [43,44], resulting in stronger, locus-specific transcripts.

During “leaky” expression, RNA polymerase II continues transcribing from actively expressed host genes into neighbouring HERV elements, passively producing chimeric host–HERV transcripts that contribute to the baseline, cell-type-specific HERV expression [45,46]. The functions of many HERV-derived RNAs remain poorly understood, although they may have important physiological roles in transcriptional regulation.

### 2.4. HERV-Mediated Host Gene Regulation

HERVs and their LTRs often act as regulatory elements involved in long-range chromosomal interactions modulating host gene expression. They act as enhancers, promoters, transcription factor binding sites, polyadenylation sites, splice sites, or RNA interference-related elements [16,21,47,48,49,50,51,52,53,54,55,56]. Many HERV/LTRs have also been co-opted as cis-regulatory elements that control the expression of embryonic developmental genes, noncoding RNAs, and immune responses. Therefore, HERV insertions are important contributors to genome evolution and form a vital integral component of host gene regulatory networks [16,50,52,53,54,56].

During embryogenesis, specific HERV envelopes mediate key developmental processes. As already noted, HERV-W encodes the fusogenic env protein syncytin-1, whereas HERV-FRD produces syncytin-2, required for trophoblast fusion in the placenta, which facilitates tolerance of the maternal immune system toward the developing foetus [16,40,57,58,59]. As demonstrated by reporter assays, HERV/LTRs also regulate neighbouring genes, providing evidence that LTR sequences could enhance transcription from nearby promoters [37,60]. Examples include HERV-9 looping to globin promoters via Nuclear Factor Y (NF-Y)/GATA binding protein 2 (GATA-2), HERV-E driving endothelin B receptor (EDNRB)/Apolipoprotein C-I (apoC-I) expression, and HERV-H serving as an alternative Gasdermin (GSDM) promoter [55,60,61].

HERVs also contribute to immune regulation, acting as endogenous sources of viral mimics that interact with Toll-like receptors (TLRs), Retinoic acid–inducible gene I (RIG-I)/Melanoma differentiation-associated protein 5 (MDA5)-Mitochondrial antiviral signalling protein (MAVS) that activate downstream type I IFN and Nuclear factor κB (NF-κB) pathways [62]. Depending on the context, this activity can enhance antiviral defence or anti-tumour immunity, drive chronic inflammation, or exert immunosuppressive effects via shared envelope domains. Certain HERV-K LTRs contain binding sites for cellular transcription factors, such as NF-κB and Activator Protein 1 (AP-1) motifs, and can drive cytokine production in positive-feedback loops that promote inflammation, which could be exacerbated by the age-associated decline in sex hormone production [35,63,64]. HERVs also modulate innate immunity through IFN-induced genes and AIM2 inflammasome activity [65]. Additionally, HERVs can generate full-length long non-coding RNAs (lncRNAs) that modulate host innate immune responses, particularly during RNA virus infections [66]. Specific HERVs, such as HERV-K18, may function as superantigens (SAgs) that activate specific T cell receptor (TCR) Vβ expressing T cells that are enriched in Tregs, which are discussed in more detail in subsequent sections [62,67]. Alternatively, HERV-K env proteins also act as HLA-presented self-antigens to shape T cell repertoires [68]. Collectively, these findings underscore the need for comprehensive genomic annotations and precise analysis of HERV cis-regulatory mechanisms to fully understand their roles in human development, immunity, and disease.

## 3. Pathophysiological Implications of HERV Activation

HERV silencing mechanisms can be compromised, allowing their reactivation in response to environmental factors including exogenous viral infections, cellular ageing, or stress (e.g., oxidative or hypoxic conditions), or via epigenetic de-repression through inhibition of DNA methylation or histone deacetylation. Under these permissive conditions, especially evolutionarily younger HERV subfamilies, such as HERV-K, HERV-W, and HERV-H, which retain open reading frames for gag, pol, and env, can produce functional viral proteins and virus-like particles (VLPs), resulting in pathogenic outcomes. Consequently, dysregulation of HERV expression can contribute to pathophysiology in various contexts, including embryogenesis and placentation [40], cancer [39,40,42,69,70,71], neurodegeneration [38], autoimmunity [72,73,74,75,76], and ageing [35], where HERV overexpression, altered splicing, or transactivation can drive cytotoxicity, immune activation, or senescence [35,50].

### Evidence of HERV Reactivation in ME/CFS

To date, only one study has documented HERV expression in ME/CFS at the locus-level, while others have relied on global PCR-based amplification methods with limited genome-association precision (Table 1). Earlier, now disproved claims of exogenous retroviruses, such as Human T-lymphotropic virus 1 or 2 (HTLV-I/II) [77], and Xenotropic murine leukaemia virus-related virus (XMRV) associations with ME/CFS have been attributed to contamination [78,79,80,81,82,83,84]. Similarly, Oakes et al. found no significant differences in HERV-K18 env transcripts or Human Herpesviruses-6 and -7 (HHV-6/HHV-7) viral loads in PBMCs between ME/CFS patients and healthy controls, with no correlation to symptom severity [85].

In duodenal biopsies analysed by immunohistology, 8 out of 12 ME patients showed the presence of HERV-K gag and env proteins of HERV-K18.1, HERV-FRD, and HERV-R, which were absent in 8 controls [86]. Interestingly, HERV protein expression co-localised with plasmacytoid dendritic cells (pDCs), implicating pDCs in ME pathology or inflammation [86]. Similarly, Rodrigues et al. reported statistically higher transcriptional activity of HERV-K env in PBMCs from moderate ME/CFS patients compared to healthy controls, suggesting a possible role in immune dysregulation that may vary with disease severity [87]. Furthermore, HERV-H, -K, and -W types were overexpressed in immune cells from patients with FM with or without ME/CFS as a comorbidity, with these increased levels of HERVs correlating with elevated IFN_β_ and IFN_γ_ levels [88]. FM symptomology overlaps with ME/CFS, with this increase in HERV expression, along with elevated cytokine levels presumed to contribute to disease pathology.

Notably, ME/CFS is often regarded as a multifactorial illness triggered by a viral (re)infection [10], although the precise mechanisms responsible for the development of ME/CFS following such an infection remain unclear. Numerous studies have explored the possibility of ongoing active infections or reactivation of latent viruses, including herpesviruses, in individuals with ME/CFS, but results have been inconsistent and remain inconclusive. Many of the viruses consistently linked to ME/CFS are herpesviruses, including EBV, HHV-6a/b, HHV-7, enteroviruses, in addition to RNA viruses, including SARS-CoV-2. Several studies have discussed the overlap between COVID-19 and ME/CFS [90,91]. SARS-CoV-2 infection increases HERV-W env antigenemia, which, together with different cytokine profiles, discriminates post-COVID from ME/CFS or FM [92]. Similarly, 3–6 months after mild/asymptomatic SARS-CoV-2 infection, saliva antibodies against EBV, HHV-6, and HERV-K are substantially increased in non-vaccinated ME/CFS patients and controls [89]. Of note, ME/CFS patients showed an overall stronger antibody response against these latent viruses, including higher IgG titres against EBV nuclear antigen-1 (EBNA1), and higher IgG responses to HERV-K, particularly in female patients [89]. Additionally, ME/CFS patients showed higher baseline EBV viral capsid antigen (VCA) IgG levels even without SARS-CoV-2 infection [89]. This suggests that even mild or asymptomatic infections can trigger or worsen latent virus reactivation, which might contribute to immune dysregulation in ME/CFS. Notably, antibody responses to infection-triggered reactivation of latent viruses are more evident and pronounced in oral mucosal samples than in plasma [89], emphasising the importance of identifying mucosal virus reservoirs, which could be sites of HERV reactivation in ME/CFS [93].

A landmark study by Giménez–Orenga and colleagues provides clear evidence of HERV dysregulation in ME/CFS. Using HERV microarrays in immune cells from a small but well-characterised, all-female cohort, the authors demonstrate distinct HERV expression profiles that differentiate ME/CFS from FM, co-diagnosed cases, and healthy controls. ME/CFS samples showed greater dysregulation, mainly involving solitary LTRs from 33 families, including HERV-H and HERV-P, compared with FM or co-diagnosed patients [13]. Within the ME/CFS group, the more severe subgroup showed greater HERV dysregulation, supporting the potential use of HERVs for patient subtyping in personalised treatments and drug repurposing trials, and as possible severity markers [13]. Although a subset of HERVs was generally downregulated across all patient groups, ME/CFS samples showed 258 upregulated and 78 downregulated HERV loci [13]. Many of these were solitary LTRs associated with or functionally linked to genes involved in pathogen sensing, T cell activation, and Th17 differentiation [13]. The more severe ME/CFS subgroup also showed coordinated immune alterations, including increased plasma cells and resting CD4 memory T cells, alongside decreased γδ T cells and altered immune response-related gene expression [13]. Importantly, activation of specific HERVs and immune-related genes positively correlated with ME/CFS diagnosis and with disease symptoms, including physical and mental fatigue [13].

Collectively, these findings suggest HERV dysregulation is a candidate molecular signature of ME/CFS, differentiating patients from healthy controls and those with overlapping comorbidities. Furthermore, locus-specific HERV upregulation links immune activation with symptom severity, supporting a possible contribution of HERVs to disease heterogeneity. Additionally, HERV dysregulation and anti-HERV immune responses in blood and tissues of ME/CFS patients suggest that HERVs may also serve as a biomarker, although variations in methodology and cohorts may explain inconsistencies. Recent microarray and loci-related studies [13] further support the potential diagnostic and mechanistic relevance of HERVs in ME/CFS. HERV expression in ME/CFS may be driven by epigenetic de-repression involving silencers like SETDB1 and TRIM28, which are amplified by infectious triggers such as SARS-CoV-2 or the reactivation of latent herpesviruses. Large-scale longitudinal studies are now required to confirm the identified dysregulated HERV signatures and to determine their role in disease initiation or progression that would underpin the development of HERV-targeted therapies for ME/CFS management.

## 4. Mechanisms of HERV Reactivation in Chronic Disease

HERV-driven chronic immune activation could contribute to chronic immune dysregulation and pathophysiology characteristic of various diseases. These include numerous cancers, such as melanoma, lymphoma, leukaemia, glioblastoma, and cancers of the pancreas, breast, prostate, ovaries, liver, kidney, cervix, oesophagus, colon, and stomach [69,70,71,94,95,96,97,98,99,100,101]. Multiple studies have shown that increased HERV expression (e.g., expression of HERV-K env upregulated in tumours compared to normal tissues) correlates with tumour progression (including migration, invasion, and growth), immune infiltration, and overall disease-specific survival and prognosis [69,70,71,94,95,96,97,98,99,100,101]. Similarly, immune-relevant HERV loci may contribute to systemic autoimmunity, including the development of type I diabetes (T1D) [73,102] and multiple sclerosis (MS) [103,104,105]. Moreover, in gastrointestinal inflammatory disorders, altered HERV expression and impaired epigenetic repression are associated with the dysregulated immune responses in Crohn’s disease, irritable bowel syndrome (IBS), ulcerative colitis and celiac disease [106,107,108,109]. How HERVs are reactivated in ME/CFS is unknown, with existing evidence limited to correlative transcriptomic profiles indicating locus-specific upregulation associated with immune dysregulation. Nonetheless, inferences can be drawn from similar conditions such as post-viral infection syndromes, autoimmune diseases (MS) and cancer, where comparable triggers, particularly virus infection and reactivation, may initiate chronic immune dysregulation leading to ME/CFS.

### 4.1. Viral Triggers Enhance HERV Expression

A plethora of viruses can activate various HERV families [110,111,112]: EBV [113], HHV-6A [114], HHV-6B [115], SARS-CoV-2 [89,116,117,118], HIV-1 [119,120,121], Adenovirus 5 [122], Human Cytomegalovirus (HCMV) [123,124], Hepatitis viruses [125,126], Dengue virus [127], Influenza A [128,129,130], and Herpes Simplex virus (HSV) [131,132]. Notably, viruses such as SARS-CoV-2 can reactivate latent viruses like EBV and HHV-6 [89,133], exacerbating dysregulation, particularly in already ill patients, such as those with ME/CFS. This could explain why 50% or more of long COVID patients meet ME/CFS diagnostic criteria [134,135,136], with these patients being more functionally impaired than the non-ME/CFS long COVID patients [137]. Virus-driven HERV reactivation could be influenced by the viral life cycle stage, host cell type, immune status, and individual differences, all of which can confound attempts to assess and identify HERV activation profiles. Overall, available data support multiple convergent mechanisms by which exogenous viruses induce HERV expression, leading to immune dysregulation, inflammation, and disease development.

#### 4.1.1. Virus Infection-Mediated Activation of HERVs

Central to HERV reactivation is virus-triggered activation of host transcription factors that activate HERV LTRs either directly or in a bystander manner. Many viral infections lead to the expression of viral proteins, including replication-related and accessory proteins that activate transcription factors such as NF-κB, AP-1, octamer-binding transcription factor-1 (Oct-1), and interferon-responsive regulators that have binding sites within HERV LTRs [121,131,132]. For example, HERV-K LTRs contain conserved binding sites for transcription factors involved in innate immunity and inflammation, including multiple NF-κB sites [63,64]. The hepatitis B virus X protein (HBx), a multifunctional oncoprotein, upregulates HERV-W env [125]. Similarly, HSV-1 immediate early protein ICP0 modulates HERV transcription through upregulating AP-1 [131], while immediate early protein 1 (IE1) activates the HERV-W LTR by increasing Oct-1 DNA-binding activity at an Oct-1 site within the LTR [132]. While viral gene products and replication-independent signalling play a critical role, full viral replication is not essential for HERV activation. For example, HCMV induces HERV expression mainly through early and late gene products rather than immediate early genes [123]. Similarly, HHV-6B can activate HERV-K18 env through receptor engagement (glycoprotein H–CD46 interaction) without requiring viral DNA synthesis [115].

#### 4.1.2. Induction of HERV-Encoded SAgs

Among virus-induced HERV products, HERV-encoded SAgs have significant immunopathogenic potential. The best-characterised example is HERV-K18 env, which encodes a SAg capable of MHC class II–dependent activation of specific T cells bearing antigen receptors encoded by specific T cell receptor Vβ genes, leading to polyclonal T-cell activation [67,138]. EBV interacts with CD21 and rapidly induces HERV-K18 env transcription in resting B cells, with the signalling mediated by latent membrane protein 2A (LMP-2A), which mimics B-cell receptor activation through its immunoreceptor tyrosine-based activation motifs (ITAMs) [113,139]. Similarly, HHV-6A and HHV-6B can induce production of HERV-K18 env independently of complete viral replication, either through interferon-mediated pathways or receptor-triggered signalling requiring de novo host protein synthesis [114,115]. This capacity of multiple herpesviruses to activate the same endogenous retroviral SAg indicates an evolutionarily convergent mechanism that subtly alters host T-cell responses, the immunological effects of such could be substantial. It is possible that minimal SAg activity can alter T-cell repertoires, promote chronic immune activation, and decrease thresholds for developing autoimmunity. This aligns with observations linking herpesvirus exposure, HERV-W and HERV-K activation, and autoimmune diseases such as MS, where HERV-W env expression correlates with HHV6-A/B antibody titres and EBV viral load [140,141].

#### 4.1.3. Immune Signalling Mediated HERV Activation

Innate immune signalling and interferon responses are another major pathway connecting viral infection to HERV expression. Viral detection via dsDNA/dsRNA pathways or by pattern recognition receptors (PRRs) elicits interferon production, which can either directly stimulate HERV transcription or foster a permissive environment for expression. Furthermore, some HERVs are integrated within host genes involved in antiviral signalling, which can influence the functioning of these pathways. For example, adenovirus infection induces HERV-K expression, which is linked to the upregulation of F-box only protein 17 (*FBXO17*), a gene encoding an intronic HERV-K sequence that negatively regulates type I interferon signalling [122]. This illustrates how HERV activation can fine-tune innate immunity during viral challenges. Similarly, HHV-6A can indirectly induce HERV-K18 through IFN-α produced by infected cells [114], as IFN-α strongly upregulates HERV-K18 expression in peripheral blood lymphocytes [138].

At the cellular level, cell type-specificity and immune context strongly influence HERV responses. Monocytes, B cells, epithelial cells, and cancer cells are sensitive to induced HERV activation, exhibiting donor-to-donor variation [115,123]. Consistent with this, env proteins of HERV-W and HERV-K are linked to proinflammatory signalling, immune cell activation, and tissue pathology, with persistent expression associated with chronic or post-acute disease states such as progressive MS and Long COVID [116,117,140,142]. In some cases, HERV activation may also enhance viral replication, as indicated by interactions between HIV-1 and HERV-K18 env [143]. Elevated HERV expression in vivo correlates with high viral loads or chronic immune activation, as seen in CMV-infected transplant recipients, HIV-viraemic individuals, hepatitis-associated liver disease, certain MS cohorts with herpesvirus serological markers, and COVID-19 patients [116,118,124,126,140,144]. Notably, HERV-FRD is highly upregulated in bronchoalveolar lavage fluid from COVID-19 patients, but not in PBMCs, with a senescence-related increase in HERV expression in human bronchial epithelial cells that may contribute to poorer outcomes in older populations [145].

#### 4.1.4. Epigenetic Regulation of HERV Expression

Apart from cellular and immune influences, epigenetic regulation constitutes a major driver of HERV activity. Viruses drive chromatin remodelling by decreasing repressive histone marks or increasing transcription factor activity, as observed for HERV-W during influenza infection [128]. Chromatin remodelling can upregulate HERV expression, thereby driving pathogenesis. This is exemplified in systemic lupus erythematosus (SLE), where hypomethylation of HERV-E clone 4-1 in CD4^+^ T cells promotes global DNA hypomethylation and IL-17–mediated inflammation via the miR-302d/Methyl-CpG binding domain protein 2 (MBD2) axis [146]. Similarly, impaired methylation of the HERV prototype HRES-1 in resting and anti-IgM–stimulated SLE B cells increases HRES-1/p28 expression [147]. Locus-specific polymorphisms (rs451401) synergise with methylation changes to elevate HRES-1/RAB4 in T cells, enhancing mechanistic target of rapamycin (mTOR) signalling upon TCR stimulation and connecting environmental stress to abnormal T cell activation in SLE [148]. Chromatin remodelling also involves dysregulation of regulatory proteins such as TRIM28 and SETDB1. Increased HERV transcription coincides with impaired expression of TRIM28, a key repressor that scaffolds SETDB1 and Krüppel-associated box zinc finger proteins (KRAB-ZFP) complexes to maintain retroviral silencing. Aberrant expression of TRIM28 and SETDB1 in inflammatory bowel disease (IBD) and related disorders (IBS, celiac disease) correlates with HERV derepression [108,109,149], driving Treg deficiency, reactive T cell expansion, and chronic immune activation, suggesting that failure to constrain HERV activity may be a common driver of persistent inflammation.

#### 4.1.5. Relevance to ME/CFS

The general principles of virus-driven HERV reactivation are relevant to ME/CFS. Many of the implicated viruses are frequently linked with ME/CFS pathophysiology. EBV, HHV-6A/B, HHV-7, adenoviruses, enteroviruses, parvovirus B19, and SARS-CoV-2 are known to induce HERV expression in other disease contexts. EBV is commonly associated with ME/CFS, establishing lifelong latency in B cells and reactivating under immune stress, leading to increased viral loads and antibody responses, including anti-EBV-dUTPase antibodies [89,150,151,152,153,154,155,156]. Similarly, HHV-6A/B, which also shows patient subgroup-specific associations with ME/CFS, displays tissue-specific reactivation [157,158,159,160,161,162]. HHV-7 activation often co-occurs with EBV, favouring enhanced immune dysregulation [158,159,161]. Additionally, SARS-CoV-2 infection often causes reactivation of EBV, HHV-6, adenovirus, and HERVs, especially in saliva, and is a major trigger of ME/CFS-like illness in long COVID [89,135,136,151,163,164]. On the other hand, adenoviruses, which persist in tonsillar and airway tissues, can activate HERV-K through interferon–modulatory pathways [122] and may serve as chronic mucosal irritants that promote broader viral and HERV reactivation in ME/CFS [164]. Enteroviruses and parvovirus B19, both implicated in ME/CFS pathophysiology [165,166,167,168,169,170], can contribute to a landscape of persistent immune activation that is conducive to HERV expression. Indeed, these virus infections or latent virus reactivation can cause increased HERV transcription in ME/CFS [88,89,92]. However, these observations remain preliminary and correlative and require more detailed and mechanistic investigations. As noted earlier, most research on viruses linked to ME/CFS has focused on blood analyses, with only a few studies investigating the mucosal virome [93]. This leaves significant questions regarding tissue-specific HERV activation and the significance and outcomes of systemic versus localised immune interactions largely unexamined.

Epigenetic alterations have also been observed in ME/CFS, including DNA methylation changes, histone modifications, and dysregulated non-coding RNAs. These often follow viral triggers (~70% of cases) such as EBV and HHV-6, which employ miRNAs and LTR transactivators to manipulate host gene expression and sustain HERV activity [171]. Several genome-wide studies have investigated the epigenetic reprogramming in ME/CFS, revealing differential methylation patterns, hyper- or hypomethylation, particularly in promoter regions regulating immune or immune-related signalling genes [172,173]. Additionally, elevated histone deacetylase activity and reduced plasma cortisol levels have also been reported [174]. Importantly, upregulated HERV loci in ME/CFS, which were discussed in Section Evidence of HERV Reactivation in ME/CFS, correlate with T-cell activation, epigenetic regulators (SETDB1, TRIM28), and shifts in immune populations, including reduced γδ T cells and increased plasma and resting CD4 memory T cells [13]. Enrichment of transcription factor binding sites in these HERVs highlights the role of epigenetic regulators in disease-related transcriptional changes. Moreover, dynamic, patient-specific methylation changes have also been observed during relapses, affecting promoters and enhancers linked to metabolic, immune, and TGF-β pathways, indicating physiological distress [175,176]. At the systemic level, these alterations seem to converge on immune, metabolic, neuronal, and endothelial pathways, also contributing to hypothalamic–pituitary–adrenal (HPA) axis dysfunction. Taken together, although there is a potential link between the activation of silenced transposable elements and the symptomatology of ME/CFS [172], more comprehensive genome-wide analyses are necessary to identify the specific loci at the single cell level and to explore the potential of HERV/LTRs in ME/CFS patient stratification and therapeutic opportunities.

In summary, the overlap between viruses associated with ME/CFS and those capable of activating HERVs supports a disease model in which initial viral triggers of ME/CFS create an environment allowing subsequent HERV reactivation. Research indicates that receptor-mediated signalling, stress responses, or the synthesis of new host or viral proteins is sufficient to induce HERV transcription. A bidirectional interaction between virus and HERV offers a plausible mechanistic link between post-infectious viral exposure and the ongoing, immune-mediated pathology observed in ME/CFS.

### 4.2. HERV-Mediated Immune Dysfunction

Activated HERVs and their LTRs have widespread effects on innate and adaptive immunity, which can be categorised into distinct but overlapping mechanisms. These include innate immune sensing, inflammatory amplification via LTRs, superantigen activity, immune suppression and modulation of antiviral restriction, and chronic immune dysregulation across various disease contexts.

Several HERV env proteins act as direct activators of innate immune receptors, functioning in a manner analogous to pathogen-associated molecular pattern molecules (PAMPs). The most extensively characterised example is the surface subunit of HERV-W env, which binds CD14/TLR4 on monocytes and dendritic cells [177]. This interaction elicits production of proinflammatory cytokines, including TNF-α, IL-6, and IL-1β and promotes dendritic cell maturation [177]. TLR4 activation by HERV-W env further drives T helper 1 (Th1) polarisation, characterised by high IFN-γ secretion [177]. Similarly, HERV-W env binds to TLR4 on pancreatic β-cells in T1D, inhibiting insulin secretion, reducing viability, and downregulating pancreatic and duodenal homeobox 1 (PDX-1)/V-maf musculoaponeurotic fibrosarcoma oncogene homologue A (Maf-A) transcription factors, while upregulating NF-κB/myeloid differentiation factor 88 (MyD88)/ TIR-domain-containing adapter-inducing interferon-β (TRIF) signalling [73]. This promotes the recruitment of macrophages to the pancreas, stimulates proinflammatory cytokines (TNF-α, IL-6, IL-12) in monocytes, promotes Th1 differentiation by dendritic cells, and triggers SAg-like T-cell responses that drive autoimmunity [73]. The expression of HERV-W env is increased in serum, PBMCs and pancreata from T1D patients [73,102,178] where HERV-W env expression correlates with macrophage infiltration [178]. HERV-W env can directly inhibit insulin secretion in human Langerhans islets by affecting β-cell viability [178]. In the central nervous system, HERV-W env activates TLR4/MyD88 signalling in glial cells, inducing TNF-α and IL-10 by inhibiting the negative regulator MyD88s [179]. In gliomas, HERV-W env expression can be detected in glioma cells as well as microglial and myeloid cells within the tumour microenvironment, where it promotes microglia-dependent secretion of proinflammatory cytokines (TNF-α, IL-6, IL-1β) and other cytokines (MCP-1, CSF-1) which promote tumour cell clustering and migration [180]. In SLE, the endogenous retroviral sequence HRES-1/Rab4 drives abnormal mTOR activation in T cells, connecting environmental stress, epigenetic dysregulation, and immune dysfunction [148]. HERV-E further enhances Th17-mediated inflammation through DNA hypomethylation and IL-17 signalling in SLE [146]. Therefore, HERV env-mediated skewing of immune responses provides a mechanistic link between HERV-W expression and chronic inflammatory or demyelinating diseases, including MS.

HERV SAgs, particularly associated with HERV-K18 env, have been implicated in multiple autoimmune diseases [181,182,183,184] with associations between specific HERV-K18 haplotypes and susceptibility to autoimmune conditions being identified [185]. Elevated HERV-K18 SAg levels have been reported in juvenile rheumatoid arthritis [183]. In T1D, HERV-K18–derived SAg activity was suspected of inducing the expansion of Vβ7^+^ bearing T cells in pancreatic islets [181,184], although this association has been challenged by later studies [73,186,187]. However, in a murine model of MS, the surface subunit of HERV-K18 env induced envelope-specific plasma IgG in immunised mice and T cell proliferation [188].

In contrast, HERVs also encode immunosuppressive activities like those of exogenous retroviruses. The transmembrane (TM) protein of HERV-K contains an immunosuppressive domain that inhibits immune cell activation by modulating cytokine release, including the induction of IL-10 in human PBMCs [189]. Furthermore, HERV-K (HML-2) env antagonises tetherin (also known as bone marrow stromal antigen 2 or BST-2), a host restriction factor that prevents the release of enveloped viral particles, by binding to tetherin without causing its degradation [190]. This enables the release of HERV-K-like particles and potentially contributes to the evolutionary persistence of this proviral family [190]. This mechanism illustrates how HERVs can modulate antiviral defences while remaining immunologically active.

Beyond protein-mediated signalling, HERV LTRs serve as inflammation-responsive regulatory elements, containing conserved binding sites for cellular transcription factors such as NF-κB [63,64]. Additionally, HERV-K (HML2) env strongly induces transcription factors ETV4, ETV5, and EGR1, which are downstream effectors of the MAPK ERK1/2 pathway and are linked to cellular transformation in breast cancer [191]. In amyotrophic lateral sclerosis (ALS), IgG antibody responses against HERV-K env correlate with disease severity, and are associated with increased env glycoprotein levels in B cells and NK cells, as well as HERV peptide-induced cytokine and chemokine shifts [192]. These include heightened IL-6 and IFN-γ production by B cells and changes in MIP-1α, MCP-1, and TNF-α secretion by CD8^+^ T cells, indicating ongoing immune activation [192]. On the other hand, pro-inflammatory cytokines like TNF-α and IL-6 can also activate the same transcription factors, creating autocrine loops that upregulate HERV-K [64]. Together, these findings suggest that HERVs promote maladaptive shifts in immune cell function rather than resolving inflammation.

#### Relevance to ME/CFS Immune Dysfunction

Collectively, the literature demonstrates that ME/CFS is characterised by persistent immune dysfunction that involves impaired cytotoxic lymphocyte responses, altered immune cell profiles, chronic immune activation, immune exhaustion and immune metabolic abnormalities [193,194,195,196,197]. Reduced NK cell cytotoxicity has been reported in several independent studies with abnormal frequencies of CD56 bright CD16^−^ NK cells, altered expression of activation and receptor molecules, and low cytotoxicity often associated with symptom severity [193,198,199,200,201,202]. Parallel impairments are also observed in CD8^+^ T-cell mediated cytotoxicity, with altered expression of adhesion molecules, receptors, perforin and granzyme B, and reduced mitochondrial membrane potential and glycolytic capacity, indicating defective effector function and impaired metabolic fitness [198,203,204,205]. Other studies report altered T-cell homeostasis, including altered CD4:CD8 ratios, reduced proliferative responses, expansion or contraction of effector memory subsets, and abnormalities in mucosal-associated invariant T (MAIT) cells [204,206,207,208]. Additionally, abnormalities of regulatory T-cells (Tregs) have also been reported, with both increased and decreased frequencies and altered forkhead box P3 (FOXP3) expression, suggesting defective immune regulation and loss of immunological homeostasis [198,200,206,209]. These findings indicate impaired cytokine production and cytotoxic function, supporting the presence of chronic antigenic stimulation or immune dysregulation.

Importantly, immune abnormalities in ME/CFS may extend beyond lymphocytes, as emerging single-cell-based evidence reveals monocyte and platelet dysregulation correlating with disease severity, implicating vascular–immune interfaces in ME/CFS pathophysiology [210]. Cytokine studies also consistently demonstrate an altered inflammatory signature, including elevations in IFN-γ, TNF-α, IL-1β, IL-6, IL-10, IL-17, and multiple chemokines that correlate with disease severity, indicating a dynamic and evolving immunopathology [195,211,212,213]. Transcriptomic and proteomic studies further support this state of immune suppression that appears to coexist with immune activation, revealing downregulated interferon signalling, altered immunoglobulin gene expression, and enrichment of inflammatory and stress–response pathways [194,214,215].

Notably, immune dysfunction in ME/CFS is tightly coupled with metabolic failure: PBMCs, NK cells, and T cells display reduced glycolysis, impaired oxidative phosphorylation, altered fatty-acid oxidation, reduced ATP production, and mitochondrial structural and signalling defects, which ultimately leave immune cells unable to meet energetic demands during activation and stress [195,197,205,216,217,218,219,220]. These metabolic constraints closely resemble profiles of immune exhaustion and senescence, supported by increased immune checkpoint proteins such as programmed cell death protein 1 (PD-1), cytotoxic T-lymphocyte-associated antigen 4 (CTLA-4), lymphocyte-activation gene 3 (LAG-3), altered CD28/CD57 expression, and transcriptional programmes associated with exhausted T cells [194,195,197,221]. Accordingly, mitochondrial dysfunction in immune cells may promote exhaustion, while exhausted or chronically activated immune cells further exacerbate damage through sustained oxidative stress and inflammation in a bidirectional loop [197].

Severity-stratified studies in ME/CFS also reveal distinct immune phenotypes, in which milder disease is associated with early immunosenescence and enhanced cytotoxic effector molecule expression, whereas severe ME/CFS is characterised by sustained T-cell activation, heightened pro-inflammatory cytokine production, and general dysregulation of T cells, NK cells, and MAIT cells [208]. Collectively, these findings support a model in which ME/CFS involves a severely compromised immunomodulatory system characterised by defective immune regulation, chronic inflammatory signalling, immune exhaustion/senescence, and an interwoven failure to regulate cellular energy metabolism, thus preventing immune cells from restoring or maintaining homeostasis. These findings also emphasise the importance of patient stratification in both mechanistic studies and clinical management.

Nevertheless, the potential contribution of HERVs to immune and metabolic dysregulation in ME/CFS remains underexplored. The persistent expression of HERV/LTRs that activate TLR4-mediated innate sensing (e.g., HERV-W env), interferon-inducible SAg activity (e.g., HERV-K18), or LTR-driven inflammatory feedback loops may result in low-level, continuous immune stimulation as in chronic viral infection, even in the absence of exogenous viral replication. This type of chronic antigen stimulation could lock T and NK cells into a dysfunctional state, where they display phenotypic changes associated with anergy, exhaustion, or senescence [193,195], resulting in immune system exhaustion and impaired antiviral defences. Taken together, ME/CFS immune abnormalities support a model where chronic antigenic stimulation that includes HERV elements drives sustained innate and adaptive responses leading to progressive immunometabolic reprogramming, which links HERV-associated pathways to ongoing dysfunction. Further studies are now needed to determine if HERVs are primary drivers or secondary amplifiers of immune dysfunction in ME/CFS.

### 4.3. HERV Reactivation Amplifies Neuroimmune Pathology

HERV activation in the central nervous system (CNS) and its contribution to neuropathology is another aspect of HERV biology with relevance to ME/CFS. Reactivation of HERVs, especially HERV-W and HERV-K, has become increasingly associated with neurological and psychiatric disorders, including MS, ALS, schizophrenia, and epilepsy. In these conditions, HERV-encoded env proteins may function as innate immune agonists, triggering neuroinflammation through TLR-mediated signalling and microglial activation [38]. Large-scale transcriptomic analyses reveal extensive expression and genetic regulation of HERVs in the adult human cortex, implicating them as contributors to psychiatric and neurological susceptibility [222]. At the tissue level, HERV activation correlates with neuroinflammatory and neurodegenerative pathology. In gliomas, HERV-W env expression in microglial and myeloid cells drives the release of proinflammatory cytokines and chemokines, which promote tumour cell clustering and migration [180]. In ALS, increased HERV-K transcripts are detected in brain tissue, particularly the motor cortex, where it strongly associates with transactive response DNA binding protein 43 kDa (TDP-43) pathology, linking HERV-driven immune activation to neurodegeneration [223,224]. HERV-K env protein is also neurotoxic, and its expression in mice induces motor neuron degeneration and progressive motor dysfunction, potentially through disruption of nucleolar function [224]. HERV-W env similarly exerts potent immunopathological effects in the CNS. It activates TLR4/MyD88 signalling in microglia and astrocytes, inducing TNF-α, IL-6, and IL-1β while inhibiting negative regulatory pathways such as MyD88s, thus maintaining glial cell activation [179].

Amongst neurological diseases, MS shows the strongest evidence for HERV-W pathogenicity. HERV-W mRNA and protein are found in peripheral blood, cerebrospinal fluid, brain tissue, active lesions, macrophages, and microglia of MS patients, with expression linked to inflammatory activity and demyelination [104,225,226,227]. Although a transcriptomic analysis reports similar HERV-W expression patterns in MS and healthy brains [228], protein-level data and disease-stage-specific associations support a pathogenic role, particularly in relapsing and progressive MS [140,141]. Alternatively, HERV-H, HERV-K18, HERV-E, RTVL, HERV-K10, and HERV Fc1 have also been associated with MS, although to a lesser extent [103,226]. Several herpesviruses, including EBV and HHV-6, have also been linked to the transactivation of HERV-W and K in MS [105,140,141]. Mechanistically, HERV-W env activates TLR4 signalling in MS lesions, both in microglia and in adjacent TLR4-expressing oligodendroglial precursor cells, inducing proinflammatory cytokine production, nitric oxide synthase activity, and nitrotyrosine formation [229,230]. These processes promote neurotoxic microglial activation, reduce myelin protein expression and clearance, impair oligodendrocyte differentiation, and ultimately cause axonal damage and remyelination failure [38,229,230], explaining the poor efficacy of immunomodulators against neurodegeneration [38]. Importantly, a phase 2b double-blind trial of temelimab, a monoclonal antibody neutralising HERV-W env, failed to reduce acute inflammation in relapsing-remitting MS but showed promising anti-neurodegenerative effects [231]. This supports the role of HERV-W in neurodegenerative processes and raises the possibility of similar HERV-mediated mechanisms being relevant in ME/CFS.

#### Relevance to Neuroimmune Pathology in ME/CFS

ME/CFS is classified as a neurological disorder characterised by cognitive impairment, psychomotor slowing, sleep disturbance, sensory hypersensitivity, pain, and dysautonomia [232,233,234]. These features indicate central regulatory dysfunction rather than isolated peripheral pathology. Neuroinflammatory models of ME/CFS focus on chronic glial activation, blood–brain barrier (BBB) disruption, and dysregulated neuroimmune signalling, especially involving the hypothalamic paraventricular nucleus and brainstem regions [232,233,234]. Peripheral immune stress caused by viral infection/reactivation and persistence, mitochondrial dysfunction, with episodic cytokine surges repeatedly activating microglia and astrocytes through neurovascular pathways, leads to relapse–recovery patterns typical of ME/CFS and Long COVID. Within this framework, HERV reactivation is a plausible molecular amplifier connecting peripheral immune activation to ongoing CNS inflammation. Consistent with this model, loss of heterochromatin in mice causes HERV reactivation, chronic neuroinflammation, and cognitive decline, showing how failed epigenetic control of HERV could drive CNS dysfunction [235]. HERV envelope proteins, particularly HERV-W env, can serve as MAMP mimics, activating TLR4 on glial cells and triggering the release of the proinflammatory TNF-α, IL-6, and IL-1β. This further exacerbates BBB dysfunction and neuroinflammatory signalling. Chronic HERV expression, driven by, for example, interferon signalling, epigenetic stress, or viral reactivation, can enhance immune signals entering the CNS, maintaining limbic and brainstem inflammation, autonomic dysregulation, and impaired remyelination. Supporting this, increased expression of HERV loci has been detected in ME/CFS peripheral blood samples mirroring symptom severity, alongside TNF-α–driven inflammatory signatures and compensatory upregulation of NF-κB inhibitory pathways [13]. Furthermore, elevated levels of IL-8 and TNF-α, along with negative regulators of NF-κB (e.g., NF-kappa-B inhibitor alpha or *NFKBIA* that encodes IκBα and TNF alpha-induced protein 3 or *TNFAIP3* encoding A20), suggest a cycle in which inflammation fails to resolve [233]. This persistent low-level NF-κB activity continuously depletes cellular resources, sustains metabolic stress, and epigenetically contributes to immune exhaustion, reflecting ME/CFS characteristic PEM intolerance and inflammatory flares, without evident tissue damage.

HPA axis dysfunction in ME/CFS most likely results from downstream effects of chronic immune–neuroinflammatory and oxidative/nitrosative stress, or infection-related immune activation. In ME/CFS, increased oxidative and nitrosative stress (O&NS), decreased antioxidants (e.g., zinc, coenzyme Q10), and chronic immune activation damage lipids, proteins, and DNA, which can lead to autoimmune responses against neo-antigens, including NO-modified epitopes driven by heightened iNOS activity [236]. Sustained cytokine signalling, such as TNF-α, redox damage, and impaired glucocorticoid receptor sensitivity, suppresses hypothalamic corticotropin-releasing hormone (CRH) output, producing characteristic HPA hypoactivity, reinforcing a self-perpetuating loop that drives fatigue, post-exertional malaise, pain, and cognitive dysfunction [236]. Alternatively, as observed in a subgroup of ME/CFS patients, high Treg levels and increased IL-10/TGF-β1 activation can cause hypocortisolism and HPA axis hypoactivity [236], which can suppress effector immunity and is linked to fatigue severity. HPA axis dysfunction can also occur following viral infection/reactivation, or independently of immune activation [236]. Yet any or all these pathways can produce chronic cycling between HPA hypoactivity and immune–inflammatory pathways.

Taken together, evidence from different neurological diseases supports a model in which HERV reactivation, particularly HERV-W and HERV-K, acts as a potent amplifier of neuroimmune pathology. In ME/CFS, HERVs can activate glial cells, cause cytokine imbalances, oxidative stress, and HPA axis suppression once neuroinflammation is established, contributing to disease chronicity, impaired recovery, and relapsing symptom patterns without the need for ongoing external infection.

### 4.4. HERV Re-Activation and ME/CFS Pathophysiology: A Hypothesis

In ME/CFS, HERVs (such as HERV-W and HERV-K families) may act as endogenous amplifiers of ongoing immune and neuroinflammatory pathology following exposure to viral and non-viral stressors. We hypothesise that acute or persistent virus infections historically associated with ME/CFS (i.e., EBV, HHV-6A/B, HHV-7, adenoviruses, enteroviruses, parvovirus B19, and SARS-CoV-2) initiate HERV derepression. In parallel, non-viral triggers including autoimmunity and chronic inflammation, oxidative and cellular stress (i.e., mitochondrial dysfunction, oxidative stress, and DNA damage), cancer-associated cellular stresses (hypoxia, altered chromatin regulation, and inflammatory tumour microenvironments), and pharmacological triggers (such as epigenetic or immunomodulatory drugs) may converge on the same inflammatory and epigenetic control pathways. Together, these triggers can cause locus-specific HERV de-repression involving viral transactivator proteins, interferon-driven innate immune signalling, NF-κB/AP-1/Oct-1 activation, and disruption of epigenetic silencing (e.g., reduced DNA methylation, loss of H3K9me3, and impaired SETDB1/TRIM28 repression) [64,131,132,171]. These epigenetic alterations lead to derepression of silenced HERV loci and increased LTR-mediated enhancer activity. This de-repression could also be shaped by host immune state, affected cell type, and inter-individual genetic and epigenetic variability, leading to persistent or recurrent expression of HERV LTRs and proteins, including SAg, even in the absence of productive viral replication [111,115,123].

Once expressed, HERV products contribute to a state of low-level chronic antigen stimulation and immune dysregulation through multiple non-exclusive mechanisms: (i) direct innate immune activation via TLR4/CD14 signalling (e.g., by HERV-W env) occurs in various cell types, including monocytes, dendritic cells, microglia, and astrocytes, leading to the production of proinflammatory cytokines such as TNF-α, IL-6, IL-1β, and iNOS. This strengthens NF-κB–dependent inflammatory responses [177,179] and eventually contributes to T/NK cell dysfunction and senescence; (ii) interferon-inducible SAg activity (e.g., HERV-K18 env) leading to MHC class II–dependent skewing and chronic activation and exhaustion of specific T cell receptor Vβ expressing T cells, similar to that described in EBV-associated autoimmunity [67,113,114]; and (iii) LTR-mediated enhancer activity that dysregulates adjacent immune and metabolic genes, maintaining low-grade inflammation and immune–metabolic stress [42,63,233]. Increased mitochondrial stress and impaired cellular metabolism further exacerbate these inflammatory feedback loops.

Alternatively, peripheral immune activation and oxidative stress cause neurovascular dysfunction. This leads to increased BBB permeability, allowing cytokines and viral or HERV-derived proteins to access the CNS. In the CNS, HERVs (such as HERV-W env) contribute to microglial priming and astrocytic activation via TLR4/MyD88 pathways, paralleling mechanisms in MS, also leading to impaired remyelination, altered neuronal signalling, BBB disruption, O&NS, impaired oligodendroglial support, and sustained neuroinflammation in limbic and brainstem regions, which have been implicated in ME/CFS neuropathology [229,230,232,233]. HERV-driven ongoing immune–glial activation [235] could indirectly suppress the function of the hypothalamic paraventricular nucleus (PVN) and HPA axis signalling by inhibiting the release of CRH from the hypothalamus and the secretion of adrenocorticotropic hormone (ACTH) from the pituitary, resulting in reduced adrenal cortisol output and impaired stress-induced HPA axis responsiveness. This cortisol deficiency hampers recovery following physiological or cognitive stressors, leading to symptom relapse and reinforcing fatigue, PEM, pain, autonomic dysfunction, sensory hypersensitivity, and cognitive impairment [236]. Thus, these combined processes create self-reinforcing cycles of HERV reactivation, inflammation, neuroendocrine dysfunction, and relapse–recovery dynamics, culminating in PEM and persistent chronic disease.

**Figure 1:**
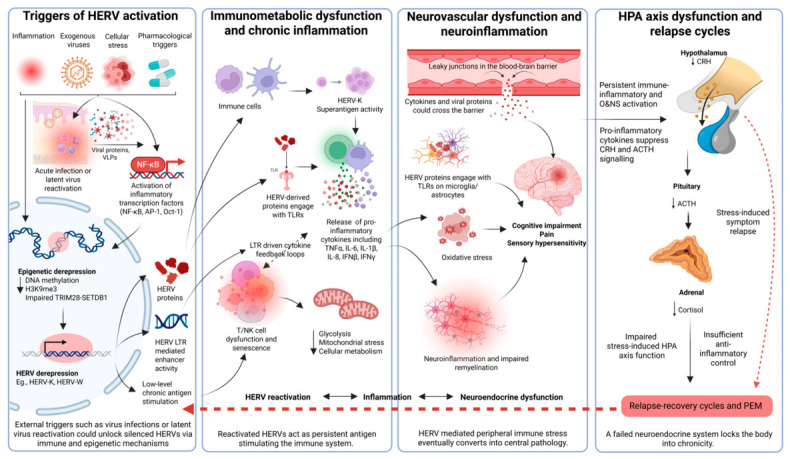
A proposed model of HERV-driven immunometabolic, neuroinflammatory, and neuroendocrine dysfunction in ME/CFS. Viral infection, inflammation, cellular stress, and pharmacological factors initiate epigenetic derepression and transcriptional activation of silenced HERV loci (e.g., HERV-K/W) in both immune and non-immune cells (including in mucosal sites), leading to the production of HERV proteins and viral-like particles (VLPs). The release of viral proteins and VLPs acts as persistent antigens and TLR ligands, driving chronic immunometabolic dysfunction, T/NK cell senescence, and pro-inflammatory cytokine release. Increased BBB permeability allows systemic mediators and HERV proteins to trigger microglial activation and neuroinflammation, which manifest as cognitive and sensory symptoms. Concurrently, persistent inflammatory signalling suppresses the HPA axis (CRH/ACTH inhibition), resulting in hypocortisolism and impaired stress responsiveness. These intersecting processes create self-reinforcing cycles of HERV reactivation, inflammation, neuroendocrine dysfunction, and relapse–recovery dynamics, culminating in PEM and persistent chronic disease. Arrows indicate the direction of activation, signalling, or feedback between processes. (Created in https://BioRender.com (accessed on 30 January 2026)).

Collectively, this hypothetical model (Figure 1) positions HERVs as stress-responsive, epigenetically activated elements that transform transient viral or inflammatory insults into self-sustaining immune exhaustion and senescence, neuroinflammation, and impaired recovery in individuals with ME/CFS. It provides a mechanistic link between the onset of post-viral infection, chronic immune activation, and CNS dysfunction in the absence of ongoing exogenous viral replication. Given that HERVs can be reactivated in a wide array of cells, including non-immune cells such as mucosal cells, it is notable that most studies to date on HERV reactivation have been conducted on peripheral blood cells.

## 5. Therapeutic and Diagnostic Outlook for HERVs

We have described how abnormal expression of HERVs/LTRs is linked to the development of various diseases. However, compelling evidence of a causal role for HERV activation in disease pathology remains elusive. Identifying disease-relevant, functional HERV loci with validated, locus-specific assays is essential, as broad-spectrum HERV detection methods cannot distinguish pathogenic activity from ubiquitous baseline retroelement expression, hindering their clinical utility as biomarkers or therapeutic targets. For HERVs to be used as clinical biomarkers, their expression must be consistent and reproducible across different cohorts and correlate with disease presence, severity, or progression, as for HERV-W in MS, and HERV-K in ALS, where targeted clinical trials are already in progress [231,237,238]. Currently, HERVs largely remain as investigational biomarkers.

The ability to utilise HERVs to differentiate between ME/CFS and FM, co-diagnosed cases, and healthy controls, identify ME/CFS-specific HERV loci linked to fatigue severity, and observe immune shifts demonstrates the potential use of HERVs as disease-specific signatures that surpass symptom-based measures [13]. Importantly, locus-specific profiling overcomes the limitations of earlier, more global HERV profiling studies that reported inconsistent results, emphasising the potential value of HERV fingerprints for diagnosis, patient subtyping, and therapeutic monitoring, including normalisation of HERV expression following rituximab treatment [239]. However, validation in larger, multi-cohort studies remains essential for clinical translation, with complementary serological and tissue-based evidence further supporting the utility of this biomarker.

Animal models provide a controlled framework to investigate the functional consequences of HERV activation and their regulatory roles in vivo. Studies across organisms, including humans, rodents, and others, demonstrate that HERV LTRs can modulate host gene expression [240,241,242]. Transgenic models offer gene-specific mechanistic insight; for example, mice expressing HERV-W env show behavioural and cognitive deficits with altered brain transcriptomes, suggesting a direct pathogenic role [243]. Animal models have also been used to study ME/CFS, typically via physical stress, immune activation, or psychological paradigms that mimic aspects of fatigue and immune dysfunction [244,245,246]. However, these models fail to capture the multifactorial nature of ME/CFS, and their reproducibility is limited by experimental design variability. Incorporating HERV biology into these in vivo models requires more refined and standardised designs that can recapitulate locus-specific HERV expression and host–immune interactions to determine if or how they contribute to ME/CFS pathophysiology.

Therapeutically, other diseases demonstrate the clinical feasibility of HERV-targeted therapies for complex chronic diseases affecting multiple organ systems. This has been investigated in MS, providing insights into the potential role of HERVs in disease pathogenesis. In MS, natalizumab decreased HERV-W env expression in PBMCs [237], while temelimab demonstrated neuroprotective effects despite limited influence on acute inflammation [231] and are currently being evaluated in clinical trials. In ALS, most patients responded to treatment with a combination of anti-retrovirals and showed reduced HERV-K levels and slower disease progression trends, supporting HML-2’s potential role in ALS pathophysiology [238]. In oncology, epigenetic therapies such as DNA methyltransferase inhibitors (azacitidine, decitabine) induce HERV expression, leading to viral mimicry, activation of dsRNA sensors (MDA5/interferon induced with helicase C domain 1 or IFIH1), type I interferon responses, and immune-mediated tumour clearance [247,248]. Additional strategies, including HERV-K–targeted vaccines, monoclonal antibodies, reverse transcriptase inhibitors, CRISPR-Cas9 approaches, and anti-HIV reverse transcriptase (RT) drugs, have shown immunomodulatory or anti-tumour effects in HERV-associated cancers [96,249,250].

**Figure 2:**
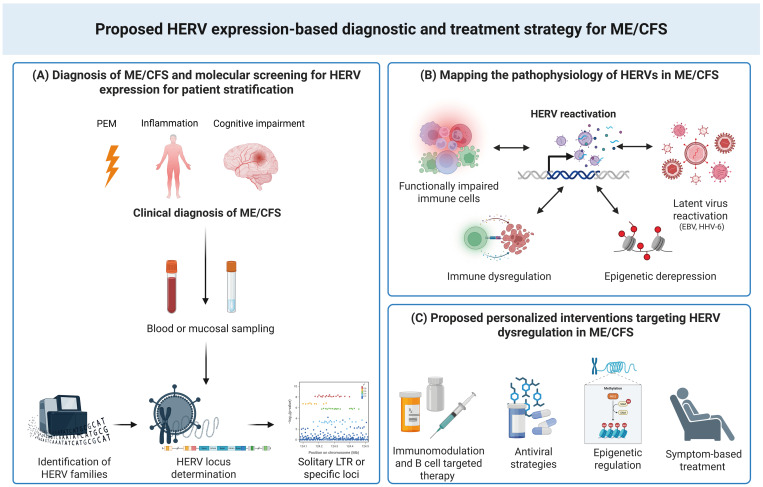
Proposed diagnosis and treatment strategy for ME/CFS based on HERV expression. (**A**) Clinical diagnosis of ME/CFS, followed by exploratory molecular screening of HERV expression in blood or mucosal samples, could enable stratification of patients into HERV-positive (HERV+) or negative (HERV−) cohorts. Patients within the HERV+ cohort could then be further evaluated to characterise the specific HERV loci involved and may potentially benefit from the proposed personalised interventions outlined in the panel (**C**). (**B**) Mapping HERV activation could uncover disease-specific profiles linked to PEM, neuroinflammation, and immune dysregulation. Isolated LTRs and HERV family-specific patterns identified in patient samples support personalised interventions, including immune-targeted therapies, antivirals, epigenetic regulators, and symptom management. (Created in https://BioRender.com (accessed on 13 January 2026)).

By contrast, ME/CFS treatment remains symptomatic, with most clinical trials focusing on downstream manifestations rather than the underlying aetiology [251]. The strong link between HERV dysregulation, immune phenotypes, and symptom severity supports a move towards mechanism-based, stratified interventions (Figure 2). Potential strategies include epigenetic modulators such as DNA methyltransferase (DNMT) or histone deacetylase (HDAC) inhibitors to restore SETDB1/TRIM28-mediated silencing of derepressed HERV loci; NF-κB antagonists to interrupt HERV-driven inflammatory feedback loops; anti-herpesvirus agents (e.g., valacyclovir) or anti-retroviral compounds (e.g., tenofovir); and senolytics to address senescence-associated secretory phenotype (SASP)-like inflammation. Diagnostic panels combining HERV expression profiles and anti-HERV serology could facilitate precision drug repurposing trials by prioritising HERV-high expressing subgroups for anti-retroviral or epigenetic therapies. Notably, a reported EBV-seropositive ME/CFS patient later diagnosed with MS showed significant clinical improvement and normalisation of HERV expression following rituximab treatment, implicating possible B-cell–HERV regulatory loops in disease modulation [239]. Such observations emphasise the importance of patient stratification in future trials.

Future research should emphasise multi-omics validation of HERV signatures, functional CRISPR-based screens to determine causality, and randomised controlled trials testing HERV-stratified interventions. Collectively, these efforts could transform ME/CFS from a syndromic diagnosis into a molecularly defined, mechanism-driven, and ultimately treatable condition.

## 6. Conclusions and Future Directions

The limited number of studies to date implicate HERVs as molecular players in the immune, metabolic and neuroimmune abnormalities observed in ME/CFS. This is evident in the identification of distinct HERV signatures that correlate with symptom severity, immune dysregulation, and disease subtypes. These findings highlight the potential of HERVs as biomarkers that can more effectively distinguish ME/CFS from related syndromes, comorbidities, and healthy states than traditional symptom-based criteria alone. Furthermore, insights gained from other HERV-associated diseases, such as MS and ALS, demonstrate the feasibility of targeting HERV-derived proteins or their epigenetic regulation to modify disease progression. Therefore, therapeutic strategies that regulate HERV activity, such as epigenetic modulators, anti-retroviral agents, or immunomodulatory interventions, offer promising mechanistic approaches that can be used alongside conventional symptomatic treatments.

Future research should focus on the mechanistic interpretation and clinical translation of HERVs’ role in ME/CFS. This includes comprehensive, multi-omics validation of HERV signatures across large, geographically diverse, and longitudinal ME/CFS cohorts to establish reproducible molecular phenotypes. Functional assays and models will help determine the causal contributions of specific HERV loci and their regulatory effects on immune, metabolic, and neuroimmune networks. Ultimately, randomised controlled trials stratified by HERV expression will be vital to assess HERV-targeted interventions, potentially turning ME/CFS from a symptom-based diagnosis into a condition defined by molecular markers that are clinically actionable.

## Figures and Tables

**Table 1:** Published studies reporting HERV activation in ME/CFS patients.

ME/CFS Diagnostic Criteria	Cohorts	Samples	Technique Used	Findings	Reference
Fukuda	39 CFS patients 9 healthy controls	PBMC	RT-qPCR	No difference in HERV-K18 env transcripts or viral copy numbers of HHV-6 and HHV-7 between CFS patients and healthy controls.	[85]
Canadian consensus criteria (CCC) and Fukuda	12 ME/CFS patients 8 healthy controls	Duodenal and stomach punch biopsies	Immunohistochemistry	8/12 duodenal samples showed HERV proteins, whereas none were detected in control samples. The observed immunoreactivity seems to be localised to pDCs. No HERV antigens detected in stomach biopsies.	[86]
Fukuda and CCC	75 moderate chronic fatigue patients (ME/CFSm) 25 severe chronic fatigue patients (ME/CFSs) 70 healthy controls	PBMC	RT-qPCR	HERV-K was overexpressed only in moderately affected individuals, but HERV-W showed no difference.	[87]
CCC and/or International Consensus Criteria (ICC)	14 female FM patients with or without ME/CFS 14 female healthy controls	PBMC	RT-qPCR	HERV-H, -K and -W overexpressed in immune cells from FM patients with or without comorbid ME/CFS, and patients with increased HERV expression also show increased levels of IFN-β and IFN-γ.	[88]
CCC	95 ME/CFS patients 110 healthy controls 50 pre-COVID plasma samples	PBMC and saliva	Immunoassay	Three to six months after mild or asymptomatic SARS-CoV-2 infection, reactivation of latent viruses, including EBV, HHV6, and HERV-K, was stronger in ME/CFS patients.	[89]
CCC and ICC	43 female subjects (8 ME/CFS patients 10 FM patients 16 co-diagnosed patients 9 matched healthy controls)	PBMC	HERV-V3 high-density microarrays	Distinct HERV expression profiles in immune cells could differentiate ME/CFS patients, fibromyalgia patients, co-diagnosed patients, and healthy controls.	[13]

## Data Availability

No new data were created or analysed in this study. Data sharing is not applicable to this article.
